# Combined bursal aspiration and corticosteroid injection for rotator cuff tear patients unresponsive to conservative management: Case report

**DOI:** 10.1097/MD.0000000000021759

**Published:** 2020-08-21

**Authors:** Dong Gyu Lee, Jang Hyuk Cho

**Affiliations:** a Department of Rehabilitation Medicine, Yeungnam University Medical Center, Yeungnam University College of Medicine; b Department of Rehabilitation Medicine, Keimyung University Dongsan Medical Center, Keimyung University School of Medicine, Daegu, Republic of Korea.

**Keywords:** bursitis, conservative treatment, rotator cuff, shoulder pain

## Abstract

**Rationale:**

Subacromial-subdeltoid (SASD) bursitis is characterized by bursal distension caused by fluid collection, commonly resulting from rotator cuff tears. Aspiration of the bursal fluid associated with rotator cuff tears tends to be overlooked. The effects of combined bursal aspiration and corticosteroid injection on full-thickness tears of the rotator cuff with SASD bursitis have not been previously reported.

**Patient concerns:**

We report the cases of 3 patients with shoulder pain caused by rotator cuff tears with marked amounts of fluid in the SASD bursa. The patients experienced intractable pain despite previous conservative management, including corticosteroid injection.

**Diagnoses:**

Physical examination and imaging studies revealed rotator cuff tears with remarkable quantities of fluid in the SASD bursa.

**Interventions and outcomes:**

The patients underwent ultrasound (US)-guided aspiration of the bursal fluid and intra-articular corticosteroid injection, following which, all patients experienced reduced shoulder pain for several months.

**Lessons:**

Combined aspiration of fluid in the SASD bursa and intra-articular corticosteroid injection in the rotator cuff tear is recommended, especially in cases with untreated shoulder pain unresponsive to previous conservative management.

## 1 Introduction

The subacromial-subdeltoid (SASD) bursa is an extra-articular sac at the shoulder girdle. This sac is separated from the glenohumeral joint by the rotator cuff.^[1,2]^ SASD bursitis is characterized by bursal distension caused by excessive synovial fluid accumulation in the bursa.^[1]^ This distended bursa commonly results from rotator cuff tears.^[3,4]^ Fluid within the SASD bursa, although nonspecific, is considered as an important sign of full-thickness rotator cuff tears.^[3,5]^ Intra-articular corticosteroid injection is widely accepted for management of rotator cuff tears with SASD bursitis.

In cases of excessive fluid accumulation in the SASD bursa, ultrasound (US)-guided diagnostic aspiration is considered the gold standard for diagnosis.^[6,7]^ However, the potential use of aspiration in the management of rotator cuff tear-associated bursal fluid accumulation tends to be underestimated.^[2]^

To the best of our knowledge, the effect of combined aspiration of considerable amounts of fluid in the SASD bursa and intra-articular corticosteroid injection for full-thickness rotator cuff tears with SASD bursitis has not been previously reported. Therefore, this case series presents 3 patients who experienced improvement in shoulder pain after combined bursal aspiration and corticosteroid injection, although they had been unresponsive to previous conservative management.

## 2 Case report

Patient I was a 73-year-old man who experienced right shoulder pain for >2 months without any history of trauma. His pain progressively worsened with a waxing and waning course, despite use of nonsteroidal anti-inflammatory drugs and supportive physical therapy. There was no obvious improvement after injection of intra-articular corticosteroids from another hospital in the previous month. He subsequently experienced limitation of activities of daily living and night pain that worsened over time and made it impossible to sleep at night. Shoulder pain on the Visual Analogue Scale (VAS) was 5 at rest and 7 during motion. There was no history of trauma, known chronic diseases, or alcohol and drug abuse.

**Figure 1:**
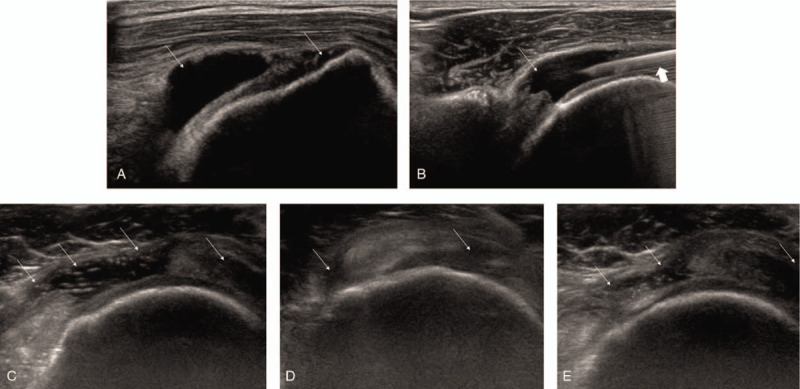
Ultrasound (US) revealing anechoic fluid collection (*thin arrow*) of subacromial-subdeltoid (SASD) bursa (A) and needle placement (*thick arrow*) within the SASD bursa (B). US revealing a large amount of anechoic fluid collection (*thin arrow*) and synovial hypertrophy of SASD bursa (C). Follow-up US 1 month later revealing diminution of the previously noted anechoic fluid (D). Follow-up US 2 month later revealing increased anechoic fluid compared to the previous US at 1 month; however, the fluid collection is slightly decreased compared to that noted in the initial US (E).

On physical examination, the skin over the shoulder joint was normal, and gentle palpation around the shoulder joint did not elicit pain. There was no loss of passive motion of his shoulder joint. However, the empty-can, Neer, and Hawkin's tests were positive. All laboratory results were within normal limits. Plain radiographs revealed a hooked acromion in the supraspinatus outlet view. US revealed marked SASD bursal effusion with a full-thickness tear of the supraspinatus tendon (Fig. 1A). To ensure an accurate diagnosis, he underwent magnetic resonance imaging (MRI) that revealed a full-thickness tear along the supraspinatus tendon with SASD bursitis.

Because the symptoms failed to resolve with conservative management, including corticosteroid injection, the authors attempted to aspirate fluid from the SASD bursa to the greatest extent possible (Fig. 1B). Under US guidance, 8 mL of clear bursal fluid was aspirated, and the specimen was sent for analysis. US-guided intra-articular corticosteroid injection was administered by penetrating the posterior joint capsule. A mixture of 0.5% lidocaine and 40 mg triamcinolone was injected into the shoulder joint space by visualizing the posterior intra-articular space.^[8]^ Distension of the joint cavity and SASD bursa after injection was checked to confirm that the procedure was accurate.

Bursal fluid analysis revealed normal white cell counts without crystals. Acid-fast bacillus stain and gram stain were negative. Fluid culture grew no microorganisms.

After aspiration and intra-articular injection, the symptoms abated. One month later, his pain score on VAS decreased from 5 to 2 at rest and from 7 to 3 during motion. Upon recheck 2 months after the combined procedure, patient I was satisfied with the pain relief; therefore, surgical management was not indicated.

Patient II was a 65-year-old man who experienced right shoulder pain for >1 month with no history of trauma and known left-sided weakness after right cerebral infarction 15 years before. He complained of unbearable pain despite corticosteroid injection 3 weeks before. Subjective pain on the VAS was 7 at rest, 9 during motion. On physical examination, empty-can and Hawkin's tests were positive. US demonstrated substantial effusion of the long biceps tendon and SASD bursa with a full-thickness tear of the supraspinatus and a partial thickness tear of subscapularis tendon. He did not want MRI for economic reasons. Under US guidance, 7 mL of the clear bursal fluid was aspirated, and a triamcinolone mixture was injected in the same way. All laboratory results, including bursal fluid, were within normal limits. Compared with the scores after 1 month, his pain score on the VAS slightly decreased from 7 to 4 at rest, from 9 to 6 during motion. Although pain abated, an additional 3 mL of bursal fluid was aspirated, and triamcinolone injected again. Upon review 3 months after the repeat procedure, his pain score on the VAS decreased from 4 to 2 at rest, from 6 to 4 during motion.

Patient III was a 70-year-old man who experienced right shoulder pain for >3 months with no history of trauma. There was no noticeable improvement following intra-articular corticosteroid injection 1 month before. Subjective pain on the VAS was 5 at rest and 8 during motion. On physical examination, the empty-can test was positive. US demonstrated substantial effusion of the long biceps tendon and SASD bursa with a full-thickness tear of the supraspinatus and subscapularis tendon (Fig. 1C). MRI revealed a full-thickness tear along the supraspinatus and subscapularis tendon with SASD bursitis. With US guidance, 9 mL of clear bursal fluid was aspirated, and a triamcinolone mixture was injected. All laboratory results, including bursal fluid levels were within normal limits. At 1 month, his pain score on the VAS decreased from 5 to 3 at rest and from 8 to 4 during motion; follow-up US revealed diminution of the previously noted bursal distension (Fig. 1D). His pain scores at 2 months were approximately at the same level as that at 1 month; follow-up US revealed increased bursal distension compared to the previous US finding but fluid accumulation was slightly decreased compared to the initial US finding (Fig. 1E).

## 3 Discussion

This case series is noteworthy because it describes, in detail, the clinical manifestations of the effect of combined bursal aspiration and intra-articular corticosteroid injection in rotator cuff tear patients who were unresponsive to previous conservative management.

The SASD bursa is an extra-articular sac at the shoulder girdle that is clinically important for the gliding movement of the rotator cuffs on the coracoacromial arch and deltoid muscle.^[1,2]^ It is separated from the glenohumeral joint by the rotator cuff; therefore, it does not usually communicate with the joint.^[1,2]^ On normal US image, the SASD bursa contains minimal fluid, surrounded by hyperechoic layers of peribursal fat, <2 mm in thickness.^[1,3,6,9,10]^

SASD bursitis is defined as inflammation of a bursa, characterized by bursal distension, caused by an excessive fluid accumulation in the SASD bursa with or without synovial hypertrophy.^[1]^ It can result from many pathological processes, commonly resulting from rotator cuff degenerative disorders, including tendinopathies or tears.^[3,4]^ Rheumatoid arthritis, gout, polymyalgia rheumatica, hydroxyapatite deposition, tuberculosis, infectious arthritis, and other pathologic conditions are less common causes.^[2–4]^ Fluid within the SASD bursa is a nonspecific sign; nevertheless, it is considered to be an important sign of a full-thickness tear of the rotator cuff tendon.^[3,5]^ Bursal distension can result from a subjacent tear where glenohumeral joint fluid may fill the SASD bursa through a full-thickness tear in the tendon.^[2]^

Blaine et al explained that inflammation in the SASD bursa can lead to shoulder pain with rotator cuff disease via inflammatory cytokines.^[11]^ These findings support the role of anti-inflammatory treatment. Furthermore, nonsteroidal anti-inflammatory drugs and corticosteroids are widely accepted treatment of inflammation in rotator cuff disease. Corticosteroids impart both anti-inflammatory and direct analgesic effects by reducing inflammatory mediators.^[12]^ The onset of action of corticosteroids is 24 to 48 hours, and their duration of action is approximately 2 to 3 weeks.^[12,13]^

In cases of substantial bursal effusion, US cannot differentiate accurately whether the fluid collection is inflammatory, infectious or hematogenous.^[7]^ Definitive diagnosis requires analysis of the fluid, and US-guided aspiration is the criterion standard.^[6,7]^ Aspiration is considered if there is clinical concern for infection; however, this tends to be overlooked in cases of bursal distension associated with rotator cuff tears.^[2]^ Nevertheless, aspiration alone may reportedly completely alleviate impingement syndrome associated with a large quantity of fluid in the SASD bursa.^[14]^

In the cases reported here, shoulder pain was caused by rotator cuff tears with SASD bursitis based on physical examination and imaging findings. All laboratory results, including analysis, stains, and cultures of bursal fluid, were normal, ruling out other causes of fluid accumulation in the SASD bursa other than rotator cuff tear. Considering that shoulder pain failed to resolve after conservative treatment, including corticosteroid injection, the authors assumed 2 possible reasons, First, the inflammation mediators remained in the large amount of bursal fluid despite of anti-inflammatory treatment; furthermore, the previous anti-inflammatory treatment might be ineffective and inflammatory pain might persist. Second, a large amount of fluid in the SASD bursa may cause mechanical pain associated with impingement of the shoulder joint. The authors attempted to aspirate fluid in the SASD bursa to the greatest extent possible, and then injected intra-articular corticosteroid under US guidance. Furthermore, there was an increase in the distension of the joint cavity and SASD bursa to confirm that the procedure was correct. Bursa cavities that had shown distension were injected with anti-inflammatory fluid mixtures.

To achieve an immediate effect, therapeutic aspiration of substantial fluid from the SASD bursa combined with corticosteroid injection may be more effective than corticosteroid injection alone. Aspiration removed the volume of inflammatory fluid, having an effect similar to that of bursectomy in reducing inflammation in the rotator cuff disease.^[11]^ Because the amount of inflammatory fluid diminished after aspiration, the effect of corticosteroid injection could be further enhanced. Nevertheless, the patients described in this report were only followed up for 3 months, and long-term follow-up was not achieved. This is a limitation of the present case series.

This report detailed the cases of 3 patients who experienced shoulder pain caused by rotator cuff tear with SASD bursitis despite conservative treatment and their successful management with combined bursal aspiration and intra-articular corticosteroid injection. Persistent bursal distension after anti-inflammatory treatment might have caused inflammatory and mechanical shoulder pain with impingement. Our patients experienced reduced shoulder pain for several months after corticosteroid injection combined with therapeutic bursal aspiration. These cases suggest that aspiration of SASD bursal fluid with rotator cuff tear is a useful practice if shoulder pain is unresponsive to conservative management. Further studies should be performed to complete the creation of a detailed treatment protocol.

## Author contributions

**Conceptualization:** Jang Hyuk Cho.

**Investigation:** Dong Gyu Lee.

**Resources:** Dong Gyu Lee.

**Writing – original draft:** Jang Hyuk Cho.
